# Supplementary material for: Ultrasound-Mediated Blood-Brain Barrier Opening Improves Whole Brain Gene Delivery in Mice
Source: Pharmaceutics. 2021 Aug 12;13(8):1245. doi: 10.3390/pharmaceutics13081245 (PMC8399273; doi:10.3390/pharmaceutics13081245)
Supplement: Supplementary file 1 [file pharmaceutics-13-01245-s001.zip › pharmaceutics-1279495-supplementary.pdf]

pharmaceutics

MDPI

Supplementary Materials: Ultrasound-Mediated Blood-Brain Barrier Opening Improves Whole Brain Gene Delivery in Mice

Marie-Solenne Felix, Emilie Borloz, Khaled Metwally, Ambre Dauba, Benoit Larrat, Valerie Matagne, Yann Ehinger, Laurent Villard, Anthony Novell, Serge Mensah and Jean-Christophe Roux

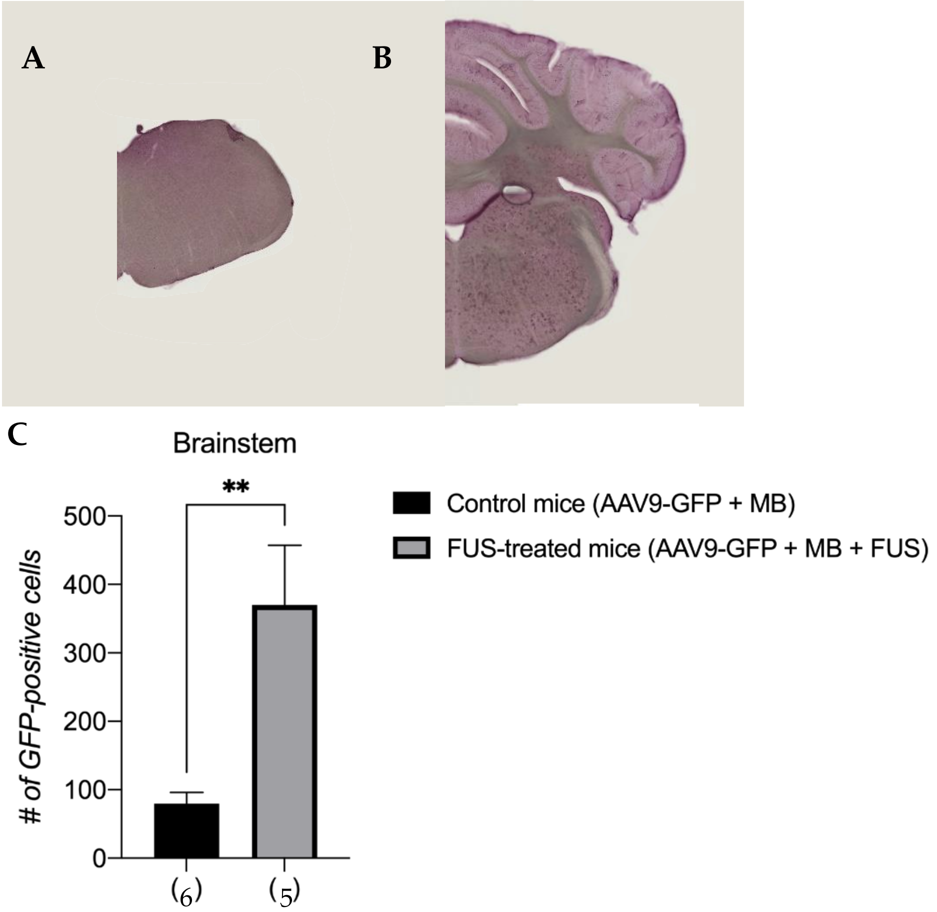

Figure S1. Immunohistochemistry using and anti-GFP antibody showed an increase in the number of GFP-positive cells in the brainstem of FUS-treated mice. Representative images with the GFP protein stained in violet are shown above the graph for control mice (**A**) and FUS-treated mice (**B**). The number of GFP-positive cells was counted using the Fiji software (**C**). Mean + SEM are represented by vertical bars, ** $p < 0.01$ Mann-Whitney rank-sum test, (n) number of animals per group.

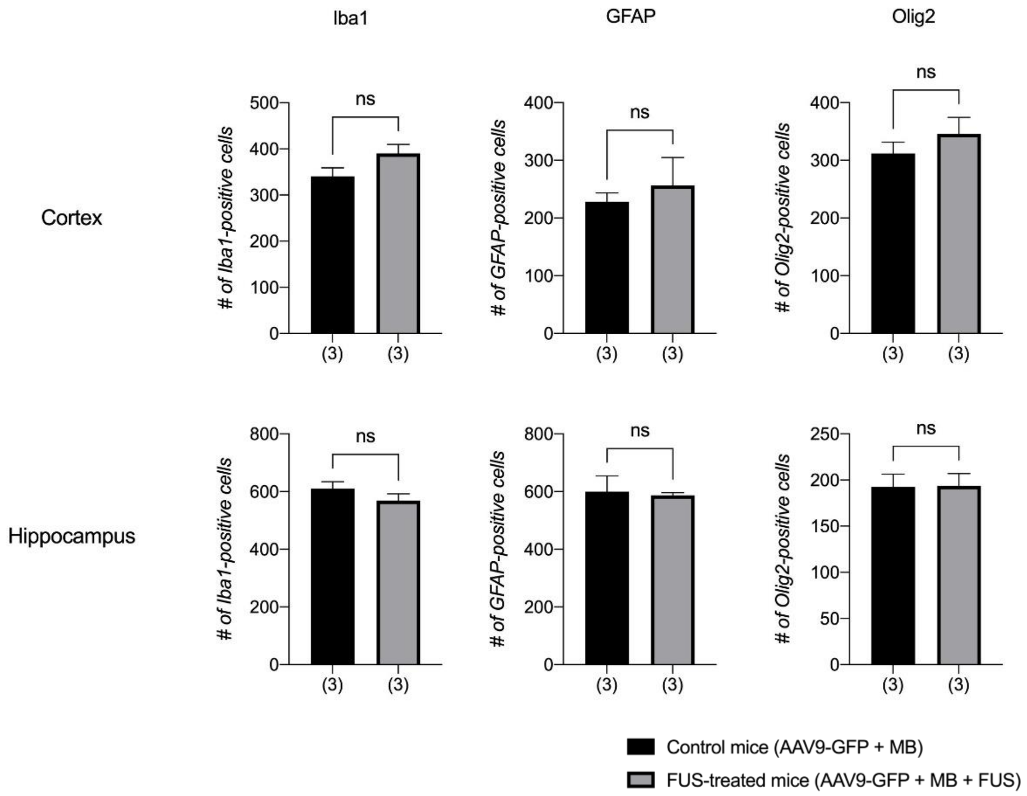

Figure S2. Immunohistochemistry using anti-Iba1, GFAP and Olig2 antibodies did not show any increase in the number of positive cells in the cortex and the hippocampus of FUS-treated mice. Mean + SEM are represented by vertical bars, ns: non-significant, Mann-Whitney rank-sum test, (*n*) number of animals per group.

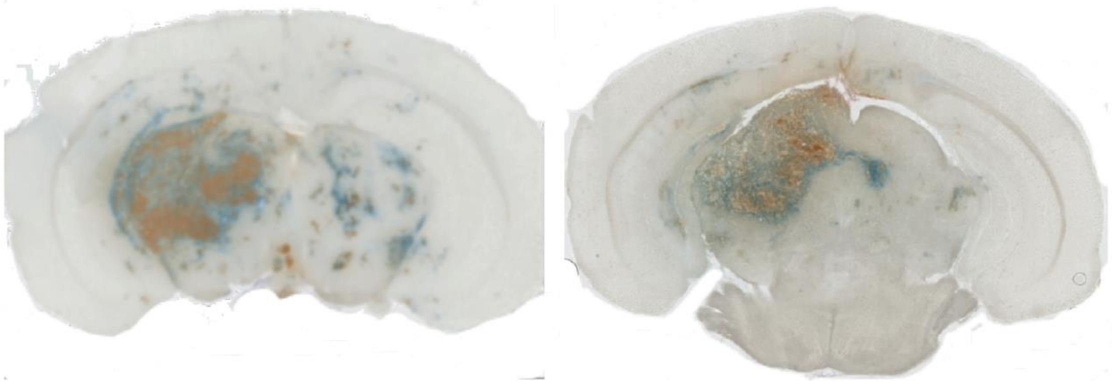

Figure S3. Important cerebral hemorrhages after FUS application at a peak negative pressure of 0.82 MPa in situ.
